# Ruptured Pseudoaneurysm of Right Hepatic Artery as Immediate Complication of Lumen‐Apposing Metal Stent Placement for Choledochoduodenostomy: A Case Report

**DOI:** 10.1155/crgm/5469497

**Published:** 2026-07-08

**Authors:** Antoine Boustany, Carson Cornell Creamer, Chidera Onwuzo, Somtochukwu Onwuzo, Rashid Abdel-Razeq, Yusuke Hashimoto

**Affiliations:** ^1^ Division of Gastroenterology, Department of Internal Medicine, University of Florida College of Medicine, Jacksonville, Florida, USA, ufl.edu; ^2^ University of Central Florida College of Medicine, University of Central Florida, Orlando, Florida, USA, ucf.edu; ^3^ HCA Florida North Florida Hospital, Graduate Medical Education Internal Medicine Residency Program, Gainesville, Florida, USA; ^4^ Department of Medicine, SUNY Upstate Medical University, Syracuse, New York, USA, upstate.edu; ^5^ Department of Gastroenterology and Hepatology, Allegheny Health Network, Pittsburgh, Pennsylvania, USA, ahn.org; ^6^ Department of Medicine, Cleveland Clinic Foundation, Cleveland, Ohio, USA, clevelandclinic.org

## Abstract

Endoscopic ultrasound‐guided biliary drainage with lumen‐apposing metal stents (LAMS) is an effective alternative for the management of malignant biliary obstruction. Despite its effectiveness, the procedure carries risks including rare but severe vascular complications. We report the first case of a ruptured pseudoaneurysm of the right hepatic artery as an immediate complication of LAMS placement and subsequent covered self‐expanding metal stent (cSEMS) placement. A 63‐year‐old female with unresectable metastatic pancreatic adenocarcinoma was admitted to our hospital with worsening abdominal pain and jaundice. Imaging revealed biliary ductal dilation and a pancreatic mass involving major vascular structures. Owing to failed endoscopic retrograde cholangiopancreatography, EUS‐guided choledochoduodenostomy with LAMS placement was performed. Immediately after LAMS deployment, massive bleeding occurred, which was controlled with an additional cSEMS. Despite the initial stabilization, subsequent gastrointestinal bleeding was observed. Computed tomography angiography (CTA) revealed a right hepatic artery pseudoaneurysm that required emergent arterial embolization. This case highlights a super rapid pseudoaneurysm formation following rescue cSEMS placement for the bleeding caused by LAMS placement.

## 1. Introduction

Endoscopic ultrasound (EUS)–guided biliary drainage with lumen‐apposing metal stents (LAMS) has emerged as an effective alternative for managing malignant biliary obstruction (MBO). It is a minimally invasive approach for biliary decompression, particularly in patients who fail endoscopic retrograde cholangiopancreatography (ERCP) [1]. EUS‐guided choledochoduodenostomy (EUS‐CDS) with LAMS has been performed as a salvage procedure in patients with distal malignant biliary obstruction (DMBO), demonstrating high technical and clinical success rates with acceptable adverse event rates [2]. Despite its advantages, EUS‐guided biliary drainage using LAMS carries non‐neglectable risks, including bleeding, stent migration, stent occlusion, perforation, and, in rare instances, vascular complications such as pseudoaneurysm formation [3]. Hepatic artery pseudoaneurysm is an uncommon but life‐threatening complication that can be iatrogenic by biliary interventions, often manifesting as delayed or massive gastrointestinal bleeding [4]. Given the rarity of this lethal complication, there is limited literature describing the early onset of pseudoaneurysm formation and its management after EUS‐CDS. We report the first case of immediate injured arterial bleeding and an acute ruptured pseudoaneurysm of the right hepatic artery as complications of LAMS placement and additional rescue SEMS placement.

## 2. Case Presentation

A 63‐year‐old female with a history of unresectable metastatic pancreatic adenocarcinoma was admitted to the Medical Intensive Care Unit (MICU) with worsening abdominal pain and marked jaundice. She had previously undergone six cycles of gemcitabine and nab‐paclitaxel but had never received radiation therapy. Computed tomography (CT) of the abdomen and pelvis performed on admission revealed a moderately distended gallbladder with multiple gallstones, intrahepatic and extrahepatic biliary ductal dilation with a common bile duct measuring 13 mm (Figure 1), and a 50–26‐mm pancreatic mass involving the celiac axis, splenic artery, portal vein, common hepatic artery, and left gastric artery. Tumoral thrombosis of the splenic vein at the confluence of the superior mesenteric vein with perisplenic varices was also noted (Figure 2).

**FIGURE 1:**
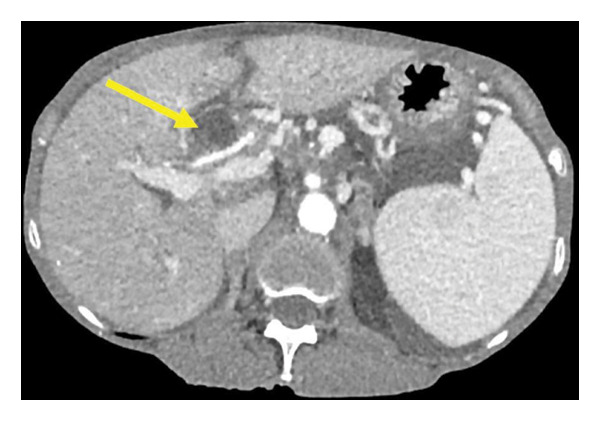
CT scan of the abdomen and pelvis with IV contrast demonstrating intrahepatic and extrahepatic biliary ductal dilation with CBD measuring 1.3 cm (yellow arrow).

**FIGURE 2:**
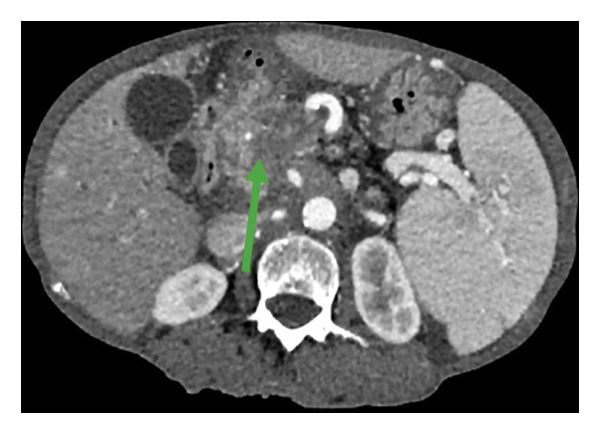
CT scan of the abdomen and pelvis with IV contrast demonstrating a 2.7 × 5.0 × 2.6‐cm pancreatic mass (green arrow) involving the celiac axis, splenic artery, portal vein, common hepatic artery, and left gastric artery.

EUS demonstrated a 16‐mm dilation in the proximal common bile duct, and the distal common bile duct was obstructed by a pancreatic head mass. ERCP was attempted, but biliary cannulation failed because of an invisible ampulla complicated by swollen duodenal mucosa. A 6‐mm × 10‐mm LAMS was successfully placed for choledochoduodenostomy after identifying a favorable puncture window in the color Doppler mode. Immediately after LAMS deployment, massive bleeding was seen through the LAMS, and the patient became hypotensive. Subsequently, a 60 × 10‐mm cSEMS was deployed through the LAMS to create a tamponade effect by compressing the bleeding spot and successfully achieving hemostasis (Figure 3). The patient was hemodynamically stabilized, intubated, and transferred back to the MICU.

**FIGURE 3:**
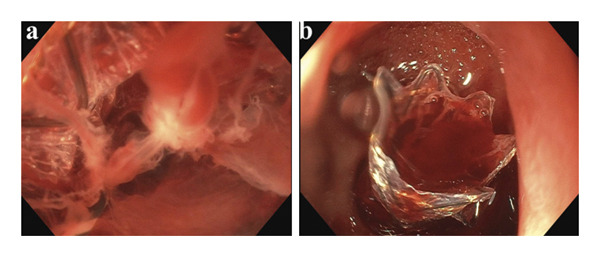
Endoscopy showing that the LAMS was placed transduodenally for choledochoduodenostomy as the first stent (a), and a covered self‐expanding metal stent was deployed through the LAMS for hemostasis as the second stent (b).

While monitoring the patient in the MICU after the procedure, recurrent bleeding was observed in the nasogastric tube. An urgent CTA performed 12 h after the procedure revealed active arterial extravasation adjacent to the cSEMS, arising from the proximal right hepatic artery, consistent with pseudoaneurysm formation (Figure 4). The patient underwent successful coil embolization of the right hepatic artery by interventional radiology. However, placement of an intravascular stent across the feeder artery was not feasible because of anatomical vascular tortuosity, cavernous transformation, and restless movement of the patient during the procedure. The patient eventually became hemodynamically stable. Given the extensive and progressive pancreatic malignancy, the patient and her family opted for comfort‐focused care over aggressive interventions. The patient was transferred to an inpatient hospice for continued symptom management and end of life care.

**FIGURE 4:**
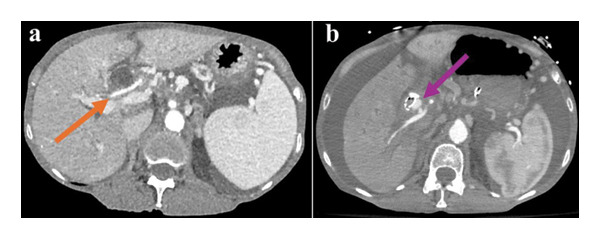
CT scan of the abdomen and pelvis with intravenous contrast performed on admission before the procedure demonstrating an intact right hepatic artery (orange arrow) adjacent to the dilated CBD (a). CTA GI bleeding protocol performed 12 h after stent placement demonstrating a pseudoaneurysm of the right hepatic artery (purple arrow) adjacent to the CBD stent with evidence of retroperitoneal hematoma (b).

## 3. Discussion

EUS‐guided biliary drainage is a well‐established technique for MBO [5, 6], and LAMS is specifically designed for transmural drainage of pancreatic fluid collections [7]. EUS‐CDS with LAMS has been reported to have feasible and safe performance. Serious adverse bleeding events have been described in the literature. Gupta et al. [8] reported in a multicenter analysis that there was 9% incidence of bleeding in EUS‐CDS with cSEMS. A bleeding case was also described in EUS‐CDS with cSEMS. Ribeiro et al. reported a delayed bleeding case 3 weeks postprocedure that the cSEMS had migrated into the duodenum, and ulceration with active bleeding was seen at the choledochoduodenostomy site. The bleeding was uncontrolled on endoscopy and necessitated angiographic embolization of the gastroduodenal artery [9]. While one prospective randomized trial demonstrated that 79 patients in the EUS‐CDS with the LAMS group showed no bleeding event as a 30‐day adverse event [10]. One fatal bleeding after EUS‐CDS using LAMS was reported in this retrospective study [11]. One fatal bleeding event was seen, although this bleeding event was delayed and occurred 17 days after the stent placement. Giri et al. reported adverse events with EUS‐guided biliary drainage in a systematic review and meta‐analysis that the incidence of bleeding varied by the stent type for EUS‐CDS, and mixed plastic with metal stents occurred with a higher incidence (1.9%) than SEMSs (1.0%) and LAMSs (0.7%) [12]. Employing electrocautery, particularly cystotomes for tract dilation, has been associated with an increased risk of bleeding. This technique can cause thermal injury to surrounding tissues, leading to both immediate and delayed hemorrhagic events. However, EUS‐CDS with LAMS showed fewer adverse events of bleeding than cSEMS [12]. LAMS are designed with wide flanges on both ends, which secure the stent in place and minimize the risk of migration. A shorter stent exerts less radial force and avoids unnecessary pressure or friction on the walls of the bile duct or duodenum, thereby decreasing the risk of vascular injury.

Our case highlights the rare presentation of significant vascular complications, particularly the exceptionally rapid formation of a right hepatic artery pseudoaneurysm within 12 h of LAMS placement, immediately followed by cSEMS placement. Several factors contributed to the complexity and significance of this case, including the rarity of rapid pseudoaneurysm formation following LAMS placement. A pseudoaneurysm forms primarily due to endothelial injury, with possible contributions from turbulent blood flow and impaired clotting, conceptually echoing Virchow’s triad but focused on vessel wall disruption rather than thrombosis, especially in cancer patients. In our case, it may explain that the first bleeding occurred due to a direct artery injury by cautery in LAMS placement and the second SEMS with sustained radial force compressed the injured right hepatic artery with immediate hemostasis of the first bleeding; instead, the arterial flow blocked by the SEMS could rapidly grow a pseudoaneurysm at the injured intimal area, leading to the second massive bleeding (Figure 5).

**FIGURE 5:**
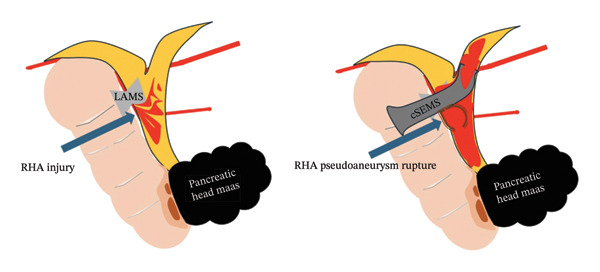
Schematic illustration of the mechanism of two different types of massive bleeding. The left picture shows massive bleeding due to RHA thermal injury after LAMS deployment. The right picture indicates ruptured bleeding from the RHA pseudoaneurysm by cSEMS blocking arterial flow into the liver.

The existing literature provides an essential context for understanding this complication, including the incidence of pseudoaneurysms, their clinical presentation and management, their association with LAMS, and the importance of early recognition. This is the first case of pseudoaneurysm of the right hepatic artery as an immediate complication of LAMS placement reported in the literature. This case highlights the risk of vascular complications after placing LAMS and emphasizes the importance of early detection and timely intervention through a multidisciplinary approach. This underscores the importance of early recognition and a multidisciplinary approach to ensuring prompt intervention. A high index of suspicion for vascular complications should be maintained after EUS‐guided stent placement. Effective collaboration among endoscopists, radiologists, and interventional radiologists is essential for timely diagnosis and intervention in cases of pseudoaneurysm formation. Rapid access to angiography and embolization services is crucial to control hemorrhage and prevent life‐threatening complications. This sequence of events is uncommon, but it highlights the need for careful vascular assessment in postprocedural bleeding and reminds us that subsequent bleeding can sometimes be more dangerous than immediate bleeding.

## 4. Conclusion

This case emphasizes the rare but serious complication of EUS‐guided biliary drainage, acute massive bleeding, and subsequent pseudoaneurysm formation following sequential LAMS and SEMS placement. Initial arterial injury likely occurred during cautery‐assisted LAMS deployment, with temporary hemostasis achieved through SEMS‐induced vascular compression. By blocking arterial flow, acute pseudoaneurysm formation and rupture can result in life‐threatening hemorrhage. Careful suspicion should be maintained for vascular complications on serial CT scans, particularly in cases with unexplained rebleeding.

## Author Contributions

Supervision: Yusuke Hashimoto; writing–original draft: Antoine Boustany; writing–review and editing: all authors.

## Funding

No funding was received for this manuscript.

## Consent

Patient consent was obtained.

## Conflicts of Interest

The authors declare no conflicts of interest.

## Data Availability

The data that support the findings of this study are available on request from the corresponding author. The data are not publicly available due to privacy or ethical restrictions.

## References

[1] Sharaiha R. Z. , Khan M. A. , Kamal F. et al., Efficacy and Safety of EUS-Guided Biliary Drainage in Comparison With Percutaneous Biliary Drainage When ERCP Fails: A Systematic Review and Meta-Analysis, Gastrointestinal Endoscopy. (2017) 85, no. 5, 904–914, 10.1016/j.gie.2016.12.023. 28063840

[2] Fugazza A. , Fabbri C. , Di Mitri R. et al., EUS-Guided Choledochoduodenostomy for Malignant Distal Biliary Obstruction After Failed ERCP: A Retrospective Nationwide Analysis, Gastrointestinal Endoscopy. (2022) 95, no. 5, 896–904.e1, 10.1016/j.gie.2021.12.032. 34995640

[3] Rana S. , Complications of Endoscopic Ultrasound-Guided Transmural Drainage of Pancreatic Fluid Collections and Their Management, Annals of Gastroenterology. (2019) Advance online publication 10.20524/aog.2019.0404. PMC6686089 31474789

[4] Kapoor R. , Agarwal S. , Calton R. , and Pawar G. , Hepatic Artery Pseudoaneurysm and Hemobilia Following Laparoscopic Cholecystectomy, Indian Journal of Gastroenterology. (1997) 16, no. 1, 32–33. 9167381

[5] Canakis A. and Kahaleh M. , Endoscopic Palliation of Malignant Biliary Obstruction, World Journal of Gastrointestinal Endoscopy. (2022) 14, no. 10, 581–596, 10.4253/wjge.v14.i10.581. 36303806 PMC9593514

[6] Rizzo G. E. M. , Carrozza L. , Rancatore G. , Binda C. , Fabbri C. , Anderloni A. , and Tarantino I. , The Role of Endoscopy in the Palliation of Pancreatico-Biliary Cancers: Biliary Drainage, Management of Gastrointestinal Obstruction, and Role in Relief of Oncologic Pain, Cancers. (2023) 15, no. 22, 10.3390/cancers15225367. PMC10670525 38001627

[7] Li J. S. , Tang J. , Fang J. , Li Z. S. , and Liu F. , Adverse Events in Endoscopic Ultrasound-Guided Choledochoduodenostomy With Lumen-Apposing Metal Stents: A Systematic Review and Meta-Analysis, Journal of Gastroenterology and Hepatology. (2024) 39, no. 9, 1769–1779, 10.1111/jgh.16614. 38736198

[8] Gupta K. , Perez-Miranda M. , Kahaleh M. et al., Endoscopic Ultrasound-Assisted Bile Duct Access and Drainage: Multicenter, Long-Term Analysis of Approach, Outcomes, and Complications of a Technique in Evolution, Journal of Clinical Gastroenterology. (2014) 48, no. 1, 80–87, 10.1097/MCG.0b013e31828c6822. 23632351

[9] Ribeiro H. , Azevedo R. , Caldeira A. , Sousa R. , Pereira E. , and Banhudo A. , Endoscopic Ultrasound-Guided Choledochoduodenostomy as Palliative Treatment: a Challenging Case Report, GE—Portuguese Journal of Gastroenterology. (2018) 25, no. 3, 146–150, 10.1159/000481175. 29761151 PMC5939861

[10] Teoh A. Y. B. , Napoleon B. , Kunda R. et al., EUS-Guided Choledocho-Duodenostomy Using Lumen Apposing Stent Versus ERCP With Covered Metallic Stents in Patients With Unresectable Malignant Distal Biliary Obstruction: A Multicenter Randomized Controlled Trial (DRA-MBO Trial), Gastroenterology. (2023) 165, no. 2, 473–482.e2, 10.1053/j.gastro.2023.04.016. 37121331

[11] Anderloni A. , Fugazza A. , Troncone E. et al., Single-Stage EUS-Guided Choledochoduodenostomy Using a Lumen-Apposing Metal Stent for Malignant Distal Biliary Obstruction, Gastrointestinal Endoscopy. (2019) 89, no. 1, 69–76, 10.1016/j.gie.2018.08.047. 30189198

[12] Giri S. , Mohan B. P. , Jearth V. et al., Adverse Events With EUS-Guided Biliary Drainage: A Systematic Review and Meta-Analysis, Gastrointestinal Endoscopy. (2023) 98, no. 4, 515–523.e18, 10.1016/j.gie.2023.06.055. 37392952

